# Parkinson’s disease and levodopa-induced dyskinesias: a quantitative analysis through ^99m^Tc-TRODAT-1 SPECT imaging of the brain

**DOI:** 10.1590/0100-3984.2023.0082

**Published:** 2024-07-24

**Authors:** Felipe Arriva Pitella, Leonardo Alexandre-Santos, Kleython José Coriolano Cavalcanti de Lacerda, Ana Carolina Trevisan, Mery Kato, Fernando Eduardo Padovan-Neto, Vitor Tumas, Lauro Wichert-Ana

**Affiliations:** 1 Nuclear Medicine and PET/CT Section, Department of Medical Imaging, Hematology and Clinical Oncology, Faculdade de Medicina de Ribeirão Preto da Universidade de São Paulo (FMRP-USP), Ribeirão Preto, SP, Brazil.; 2 Department of Psychology, Faculdade de Filosofia, Ciências e Letras de Ribeirão Preto da Universidade de São Paulo (FFCLRP-USP), Ribeirão Preto, SP, Brazil.; 3 Department of Neuroscience and Behavioral Sciences. Faculdade de Medicina de Ribeirão Preto da Universidade de São Paulo (FMRP-USP), Ribeirão Preto, SP, Brazil

**Keywords:** Parkinson disease, Levodopa, Dyskinesias, Dopamine, Tropanes, Doença de Parkinson, Levodopa, Discinesias, Dopamina, Tropanos

## Abstract

**Objective:**

To compare the dopamine transporter (DAT) density with other risk factors for L-DOPA-induced dyskinesia (LID) in patients with Parkinson’s disease (PD), with and without LID.

**Materials and Methods:**

We evaluated 67 subjects: 44 patients with idiopathic PD of varying degrees of severity (PD group), and 23 healthy age-matched volunteers (control group). Among the 44 patients in the PD group, 29 were male and the following means were recorded at baseline: age, 59 ± 7 years; disease duration, 10 ± 6 years; Hoehn and Yahr (H&Y) stage, 2.16 ± 0.65; and Unified Parkinson’s Disease Rating Scale part III (UPDRS III) score, 29.74 ± 17.79. All subjects underwent ^99m^Tc-TRODAT-1 SPECT. We also calculated specific uptake ratios or binding potentials in the striatum.

**Results:**

The DAT density in the ipsilateral and contralateral striata was lower in the PD group. The variables disease duration, L-DOPA dosage, doses per day, L-DOPA effect duration time, H&Y stage, and UPDRS III score explained the occurrence of LID. The DAT density in the ipsilateral striatum, contralateral striatum, and caudate nucleus was lower in the patients with LID than in those without.

**Conclusion:**

Our findings suggest that presynaptic dopaminergic denervation is associated with LID in individuals with PD.

## Abstract (pt)

**Objetivo:**

Comparar a densidade do transportador de dopamina (DAT) com outros fatores de risco para discinesia induzida pela L-DOPA em pacientes com doença de Parkinson, com e sem discinesias.

**Materiais e Métodos:**

Sessenta e sete sujeitos, 23 voluntários saudáveis e 44 pacientes pareados por idade com diferentes graus de gravidade da doença de Parkinson idiopática (29 homens; idade média ± desvio-padrão (DP), 59 ± 7 anos; duração média ± DP dos sintomas, 10 ± 6 anos; H&Y: média ± DP, 2,16 ± 0,65; UPDRS III: média ± DP, 29,74 ± 17,79). Todos os sujeitos realizaram SPECT cerebral com ^99m^Tc-TRODAT-1. Além disso, foram calculadas as taxas de captação específica ou potenciais de ligação no estriado.

**Resultados:**

A densidade de DAT do estriado ipsilateral ou contralateral foi menor no grupo doença de Parkinson. As variáveis duração da doença, dosagem de L-DOPA, doses por dia, tempo de duração do efeito da L-DOPA, H&Y e UPDRS III explicaram a ocorrência de discinesia. Adicionalmente, pacientes com discinesia exibiram menor densidade de DAT no estriado ipsilateral ou contralateral e no núcleo caudado do que os pacientes sem discinesia.

**Conclusão:**

O presente estudo sugere que a denervação dopaminérgica pré-sináptica na doença de Parkinson está associada ao desenvolvimento de discinesia induzida pela L-DOPA.

## INTRODUCTION

Parkinson’s disease (PD) is a progressive neurodegenerative disease affecting approximately 1% of persons over 60 years of age and up to 4% of those over the age of 80^(1)^. The diagnosis of PD commonly relies on the cardinal features of bradykinesia, rigidity, tremor, and postural instability, coupled with gradual symptom progression and a sustained response to therapy with levodopa (L-DOPA). Nonmotor symptoms, such as constipation, cardiac arrhythmias, sleep disorders, and cognitive deficits, are also observed in^(2,3)^

The main hallmark of PD is the loss of dopaminergic neurons in the substantia nigra pars compacta^(4)^. That leads to depletion of dopamine in the striatum and the accumulation of α-synuclein protein in the brain as Lewy bodies. In addition, various other mechanisms have been implicated in dopaminergic cell death in PD^(5)^: oxidative stress, excitotoxicity, mitochondrial dysfunction, neuroinflammation, protein aggregation, phosphorylation, genetic defects, and toxins.

The active ingredient in carbidopa-levodopa combination therapy—L-DOPA—remains the most effective treatment for PD, improving tremor, rigidity, and bradykinesia, particularly in the early stages of the disease. It is well tolerated, has a rapid onset, reduces the risk of death, and is the least expensive medication for this condition. Although chronic exposure to L-DOPA is commonly associated with the development of significantly disabling motor fluctuations and dyskinesias, recent findings suggest a more nuanced association. The reported incidence rates of L-DOPA-induced dyskinesia (LID) range from 9% to 80%. The condition is clinically heterogeneous and commonly presents as chorea or choreoathetosis, although myoclonus, akathisia, ballism, and other abnormal movements have also been described. It typically appears first on the side most affected by PD and in the legs before the arms. Recent studies have demonstrated that the risk factors for LID are less likely to be related to the duration of L-DOPA use and are more likely to be related to the duration of PD and the daily dose of L-DOPA^(6)^. In addition, some authors have shown evidence that presynaptic dopaminergic denervation in PD plays a role in LID^(7)^.

The underlying mechanisms for LID remain unclear. However, it has been suggested that pulsatile stimulation of the postsynaptic receptors by intermittent administration of L-DOPA leads to downstream changes in proteins and genes, causing alterations in striatal output to promote dyskinesias.

The dopamine transporter (DAT), a protein in the presynaptic membranes on the dopaminergic terminals, is considered a marker of dopamine terminal innervations and regulates extracellular dopamine concentration^(8)^. Various agents, all based on cocaine or closely related tropane derivatives, have been employed in DAT single-photon emission computed tomography (SPECT) to investigate striatal dopamine terminal function in typical and atypical PD. Reductions in the striatal uptake of those radiophar-maceuticals provide a helpful marker of functional DAT loss, which is a valuable means of supporting or rejecting a diagnosis of parkinsonism associated with striatal dopamine deficiency. Because the technetium-99m-labeled tropane derivative TRODAT-1 (^99m^Tc-TRODAT-1) binds DAT, it has been employed to evaluate the density of the transporter on imaging^(9)^.

The objective of this study of patients with PD was to compare those with and without LID, in terms of the DAT density on ^99m^Tc-TRODAT-1 SPECT brain scans. We also evaluated other risk factors for LID.

## MATERIALS AND METHODS

### Study design

The research subjects were prospectively included in two groups (control and PD). The PD group was subdivided into PD with LID and PD without LID, and the controls were matched to the patients in those subgroups regarding age, level of education, and sex. After computer-generated randomization designed to yield the groups, the data underwent blind analysis.

### Subjects

This prospective study included a total of 67 subjects: 44 patients with idiopathic PD of varying degrees of severity (PD group); and 23 healthy age-matched volunteers (control group). Among the 44 patients in the PD group, 29 were male, the mean age was 58.81 ± 7.02 years, and the mean disease duration was 9.55 ± 5.57 years (range, 1–21 years). Among the 23 subjects in the control group, 15 were female and the mean age was 58.79 ± 10.78 years. Individuals in the control group were free of moderately severe or severe dementia, PD, parkinsonian syndromes, neuropsychiatric disorders (such as schizophrenia, attention deficit hyperactivity disorder, symptomatic depression, and other conditions that could alter the dopaminergic system), as well as having no history of alcohol or drug abuse, other known organic brain lesions, stroke, claustrophobia, chronic psychotropic medication use, use of drugs known to interfere with the binding of TRODAT-1 to DAT, trauma with loss of consciousness, or smoking. A neurologist known to be an expert in extrapyramidal disease evaluated the patients. The diagnosis of PD was made on the basis of the UK Parkinson’s Disease Society Brain Bank clinical diagnostic criteria^(10)^. The study was approved by the local research ethics committee, and all participating subjects gave written informed consent.

All patients were receiving L-DOPA therapy, some as monotherapy and others as adjunctive therapy with dopaminergic agonists, amantadine, or both. Most (88.6%) of the patients had bilateral motor symptoms. None of the patients were taking medication that might have interfered with striatal ^99m^Tc-TRODAT-1 uptake. All patients were evaluated with the Hoehn and Yahr (H&Y) scale, the score on which categorizes PD from stage 1 to stage 5, and with the Unified Parkinson’s Disease Rating Scale part III (UPDRS III), which is scored from 0 to 108, higher scores indicating worse motor functioning^(11)^. All PD group patients underwent brain SPECT with ^99m^Tc-TRODAT-1^(12)^. The UPDRS score was calculated during the “on” state (i.e., 4 h after the injection of ^99m^Tc-TRODAT-1). That timing is associated with the peak plasma concentration of L-DOPA, when patients are typically experiencing their best motor function. In the PD group, the mean UPDRS III score was 29.74 ± 17.79 (range, 9–74) and the mean H&Y stage was 2.16 ± 0.65 (range, 1–4).

### Radiopharmaceutical

The radiopharmaceutical was prepared from a previously formulated lyophilized TRODAT-1 kit (Institute of Nuclear Energy Research, Lung-Tan, Taiwan). The kit was reconstituted with 1,628 MBq (44 mCi) of freshly eluted ^99m^Tc-sodium pertechnetate in 5 mL of saline solution, and the resulting mixture was incubated at 100°C for 30 min to complete the labeling. After cooling to room temperature, ^99m^Tc-TRODAT-1 with a radiochemical purity > 90% (determined by dual-strip instant thin-layer chromatography) was obtained in a neutral solution (pH 7.0–7.5).

### Image acquisition

**Figure 1:**
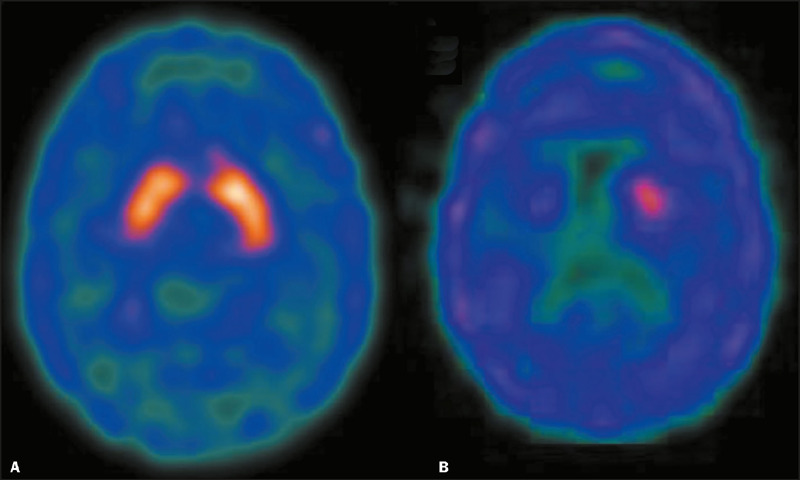
Comparative ^99m^Tc-TRODAT-1 SPECT images of a healthy individual **(A)** and a patient with H&Y stage 3 PD **(B).** The image of the patient shows significantly lower DAT density within the striatum, indicating disease progression. Notably, the limited uptake is more pronounced in the right (contralateral) striatum than in the left (ipsilateral, more symptomatic) striatum, highlighting the asymmetric nature of neurodegeneration in PD.

All SPECT brain scans were acquired with a dual-head gamma camera (BrightView XCT; Philips Medical Systems, Cleveland, OH, USA), at 4 h after injection of 740-1,110 MBq (mean, 865.8 ± 74.0) of ^99m^Tc-TRO-DAT-1. Data were acquired in a 128 × 128 matrix, with a 1.2 zoom through 360° rotation (180° for each head) at 3° intervals and 30 s per angle step. Images were reconstructed by using the iterative method and a Butterworth filter (order, 2.00; cut-off, 0.22). We obtained transverse, coronal, and sagittal slices (thickness, 4.7 mm) oriented to the orbitomeatal line. Chang’s attenuation correction was applied with a coefficient of 0.12/cm. Figure 1 shows typical ^99m^Tc-TRODAT-1 SPECT images from subjects in the control and PD groups.

### Semi-quantitative assessment

Five adjacent transaxial slices with the highest radio-pharmaceutical uptake in the basal ganglia were summed for semi-quantitative analysis of striatal specific DAT binding of ^99m^Tc-TRODAT-1 using an image analysis package (JETpack; Philips Medical Systems). Fixed regions of interest (ROIs) were drawn manually over the summed transaxial slice of each hemisphere of the striatum as a whole, the putamen, and the caudate nucleus. For the quantification of DAT binding, the orbitomeatal line was used as the projection plane for ^99m^Tc-TRODAT-1, which was therefore located above the nucleus accumbens. Consequently, our analysis focused exclusively on the body of the putamen and the head of the caudate nucleus within the striatum. In addition, an irregular ROI was drawn manually over the occipital cortex. The specific uptake ratio or binding potential index (BPI) was calculated for the striatum by subtracting the mean counts per pixel in the occipital lobe (background) from the mean counts per pixel in the striatum as a whole and dividing the result by the mean counts per pixel in the background. In the PD group, the striatum opposite the side with dominant symptoms was designated the contralateral striatum. In the control group, the left and right striata were arbitrarily designated the ipsilateral and contralateral striata, respectively. The ^99m^Tc-TRODAT-1 images were analyzed semi-quantitatively by two experienced nuclear medicine physicians who were blinded to the groups and to the clinical conditions of the subjects.

### Statistical analysis

To compare the subjects in the control group with the patients in the PD group, in terms of the BPIs in the striatum, caudate nucleus, and putamen, we employed a simple linear regression model adjusted for gender, age, and level of education. Another simple linear regression model, adjusted for the same factors, was used in order to evaluate the relationships between LID and clinical findings in the PD group. The following variables were analyzed: gender, age (in years), schooling (in years), disease duration (in years), age at symptom onset (in years), UPDRS III score, daily L-DOPA dose (in mg), number of doses per day, and L-DOPA effect duration time (in hours). To compare patients with and without LID, differences between the means of the variables were analyzed with Student’s t-tests, considering the approximate degrees of freedom. Those approximate degrees of freedom were employed to calculate the corresponding p-value for the observed t-statistic. The choice to use approximate degrees of freedom was based on the specific characteristics of the samples and the assumptions of the statistical test, with the aim of determining the appropriate approach to data analysis. Concerning the DAT BPI in the striatum, caudate nucleus, and putamen, an adjusted simple linear regression model was also used for intergroup comparisons (PD with and without LID). The statistical analysis was performed with the Statistical Analysis System for Windows, version 9.3 (SAS Institute Inc., Cary, NC, USA).

## RESULTS

The demographic and clinical characteristics of the PD group are shown in Table 1. Visual inspection frequently revealed apparent differences between the PD and control group in terms of the uptake of ^99m^Tc-TRODAT-1. The mean BPIs in the control group were 1.18 ± 0.22 in the ipsilateral striatum, 1.21 ± 0.25 in the contralateral striatum, 1.14 ± 0.28 in the contralateral putamen, 1.0 ± 0.23 in the ipsilateral putamen, 1.33 ± 0.29 in the contralateral caudate nucleus, and 1.31 ± 0.27 in the ipsilateral caudate nucleus. For the ipsilateral and contralateral striata (total and subregions), the DAT density was significantly lower in the PD group than in the control group. In addition, the limited uptake was more pronounced in the contralateral striatum than in the ipsilateral striatum. We found that the DAT density in the ipsilateral and contralateral striata and caudate nuclei was statistically lower in the patients with LID than in those without, whereas that in the contralateral and ipsilateral putamina trended lower in the patients with LID than in those without (Table 2).

**Table 1:** Demographic and clinical characteristics of patients with PD, with and without LID.

Characteristic	LIDpos (n = 21)	LIDneg (n = 23)	LIDneg – LIDpos	95% CI	P
Gender (female/male), n/n	9/12	6/17	–	–	0.940
Age (years), mean ± SD (range)	59.38 ± 5.68 (49-68)	58.20 ± 8.38 (44-73)	−1.18	−5.50 to 3.14	0.585
Years of schooling, mean ± SD (range)	6.48 ± 3.75 (2-16)	5.38 ± 4.07 (0-20)	−1.10	−3.48 to 1.28	0.356
Age at symptom onset (years), mean ± SD (range)	46.74 ± 7.64 (35-60)	53.65 ± 11.00 (33-70)	6.91	1.19 to 12.64	0.0191*
Disease duration (years), mean ± SD (range)	12.90 ± 5.04 (3-21)	6.50 ± 4.15 (1-17)	−6.4	−9.22 to −3.58	< 0.0001^†^
L-DOPA dosage (mg), mean ± SD (range)	1009.21 ± 413.09 (500-2250)	504.76 ± 333.88 (150-1200)	−504.45	−734.34 to −274.56	< 0.0001^†^
Doses per day, mean ± SD (range)	6.05 ± 1.87 (3-9)	3.76 ± 1.67 (3-8)	−2.29	−3.37 to −1.21	0.0001^†^
L-DOPA effect duration (hours), mean ± SD (range)	3.62 ± 1.80 (2-9)	5.08 ± 1.85 (2-12)	1.46	0.35 to 2.57	0.0112^†^
H&Y stage, mean ± SD (range)	2.48 ± 0.60 (2-4)	1.86 ± 0.56 (1-3)	−0.62	−0.97 to −0.27	0.001*
UPDRS score, mean ± SD (range)	35.57 ± 16.20 (13-74)	24.18 ± 17.67 (9-72)	−11.39	−21.97 to −1.11	0.0307*

LIDpos, LID positive; LIDneg, LID negative.

*P < 0.05; ^†^*P* < 0.01.

**Table 2:** DAT density in the striatum and subregions in patients with PD, with and without LID.

Region	LIDpos (n = 21)	LIDneg (n = 23)	LIDneg – LIDpos	95% CI	P
Striatum ipsilateral	0.4 ± 0.25 (0.04-1.03)	0.59 ± 0.33 (0.19-1.47)	0.19	−0.51 to 0.89	0.0362*
Striatum contralateral	0.34 ± 0.20 (0.03-0.78)	0.51 ± 0.27 (0.19-1.16)	0.17	−0.47 to 0.81	0.0216*
Putamen ipsilateral	0.33 ± 0.22 (0.03-0.96)	0.50 ± 0.36 (0.13-1.47)	0.17	−0.28 to 0.62	0.0633
Putamen contralateral	0.30 ± 0.19 (0.01-0.61)	0.44 ± 0.29 (0.08-1.10)	0.14	−0.15 to 0.43	0.0630
Caudate nucleus ipsilateral	0.50 ± 0.34 (0.01-1.20)	0.73 ± 0.33 (0.32-1.51)	0.23	−0.47 to 0.93	0.0262*
Caudate nucleus contralateral	0.42 ± 0.29 (0.02-1.30)	0.61 ± 0.29 (0.21-1.28)	0.19	−0.34 to 0.72	0.0357*

*P < 0.05.

Of the 44 patients in the PD group, 21 had LID and 23 did not. Among the independent clinical variables (Table 1), age at symptom onset, disease duration, L-DOPA dosage, doses per day, L-DOPA effect duration time, H&Y stage, and UPDRS III score were associated with LID (p < 0.05 for all).

## DISCUSSION

In the present study, we have investigated the association between DAT density on ^99m^Tc-TRODAT-1 SPECT of the brain and other risk factors for LID in PD. Our results show that DAT density in the striatum and caudate nucleus was significantly lower in patients with PD than in healthy individuals, that difference being greatest in the contralateral striatum. We have established a link between low DAT density and LID occurrence, which was found to be significantly associated with clinical variables such as the age at symptom onset, disease duration, and L-DOPA dosage. The trend toward lower DAT density in the putamina of the patients with LID suggests that the putamen plays a current but less pronounced role in the occurrence of LID than do the other regions of the striatum. This study underscores the potential of DAT as a therapeutic target in PD and should prompt further research to determine the broader implications for treatment.

The main pathological hallmark of PD is the loss of dopaminergic neurons in the substantia nigra pars compacta. Imaging the dopaminergic system with positronemission tomography (PET) or SPECT has proven valuable for understanding and diagnosing neurodegenerative diseases like PD. In patients with PD, neurons in the striatum have been imaged by using various markers of dopaminergic function. Numerous imaging studies have identified distinctions between patients with PD and age-matched healthy control subjects^(13,14,15,16,17,18)^.

Consistent with previous research, our findings suggest that the administration of L-DOPA remains the most effective treatment for controlling motor symptoms in PD^(19)^. However, although L-DOPA may significantly improve PD symptoms, its long-term use can be limited by the development of LID^(20)^. The mechanisms underlying LID remain unclear; they are observed in approximately 50% of patients within five years of the initiation of L-DOPA treatment, with various forms of presentation^(21,22)^.

Studies in animal models have shown that an “initiation effect” is central to the development of LID. Early administration of L-DOPA triggers a biochemical and transcriptional response sensitized in the striatum. It follows subsequent dopaminergic stimulation and progressive and persistent dyskinetic behaviors^(22,23)^.

Various changes in striatal function have been described as contributing to the cellular sensitization observed during LID expression. Such changes include increased glutamatergic signaling, extracellular signal-regulated kinase/cyclic AMP response element-binding protein activity, expression of L-DOPA-dependent genes, and increased translation activity^(22)^. In addition, presynaptic hypotheses attempt to explain the pathophysiology of LID by relating it to presynaptic control dysregulation of vesicular storage, as well as to uncontrolled release and decreased reuptake of dopamine^(23)^.

Although results in the literature are variable, an association has been described between LID and the uptake deficit in the striatum seen on DAT-SPECT and DAT-PET. A retrospective study of 127 patients undergoing ^18^F-FPCIT PET, who were followed for at least two years after the initiation of dopaminergic treatment, showed that radiopharmaceutical uptake in the anterior putamen, posterior putamen, and striatum was predictive of LID development, although the BPI for the striatum and caudate nucleus was not^(24)^.

One review, focused on the application of PET in evaluating the development of LID in patients with PD, showed that the uptake deficit in the putamen on ^11^C-MP PET with LID at peak dose is associated with motor fluctuations^(25)^. That finding supports the concept that a gradual loss of DAT availability may result in the loss of compensatory mechanisms when dopamine levels increase substantially after a dose of L-DOPA^(25,26)^. In a study of patients with PD who underwent ^18^F-DOPA PET^(27)^, radiopharmaceutical uptake was found to be lower in the patients with motor fluctuations than in those with a stable response to L-DOPA (12% lower in the caudate nucleus and 28% lower in the putamen). These findings indicate that reduced capacity of the striatum to store dopamine plays a role in the development of motor fluctuations.

Our findings support the presynaptic hypotheses. We observed significant differences between the PD group patients with and without LID in terms of the mean BPIs for the ipsilateral striatum, contralateral striatum, ipsilateral caudate nucleus, and contralateral caudate nucleus, all of which were lower in the patients with LID. Although the BPI for the putamen was also lower in the patients with LID, the difference was not significant. That suggests that greater presynaptic dopaminergic involvement is needed for the development of LID, given that, in PD, the uptake deficit in the striatum occurs primarily in the putamen. Another explanation for these data is the frequent uptake deficit in the putamen found in our sample, in which most patients already had bilateral motor symptomatology, although most were at an early stage (H&Y stage 2).

The clinical parameters found to be associated with LID in our sample (disease duration, age at symptom onset, L-DOPA dosage, number of doses per day, duration of L-DOPA effect, UPDRS score, and H&Y stage) might contribute to LID development through various mechanisms, including dopaminergic system dysregulation and increased dopamine availability. The same rationale applies to a DAT deficit in the striatum, which reduces the reuptake of dopamine released in the synaptic cleft. Given this scenario, the association of LID with factors beyond a low BPI in the striatum, as determined by ^99m^Tc-TRODAT-1 SPECT, suggests a multifactorial aspect in the development of LID.

One limitation of the present study was the lack of a control for disease duration in the linear regression. Given that the rate of natural dopaminergic neuron loss with aging is probably lower than the pathological loss in PD, future studies should investigate that aspect. That difference could have an impact on the quantitative analysis of DAT density in the striatum.

## CONCLUSIONS

Our findings suggest that presynaptic dopaminergic denervation is associated with LID development in PD. This is new information that has yet to be confirmed. What is well known is that LID is associated with other clinical variables, particularly disease duration. The evaluation of DAT density through the use of ^99m^Tc-TRODAT-1 SPECT could complement clinical assessment, potentially contributing to the development of new preventive strategies for LID.
